# Development of a Dispersive Liquid–Liquid Microextraction Method for Quantification of Volatile Compounds in Wines Using Gas Chromatography–Mass Spectrometry

**DOI:** 10.3390/metabo15020129

**Published:** 2025-02-13

**Authors:** Dinesha Katugampala Appuhamilage, Rebecca E. Jelley, Emma Sherman, Lisa I. Pilkington, Farhana R. Pinu, Bruno Fedrizzi

**Affiliations:** 1 School of Chemical Sciences, University of Auckland, 23 Symonds Street, Auckland 1010, New Zealand; dkat036@aucklanduni.ac.nz (D.K.A.); lisa.pilkington@auckland.ac.nz (L.I.P.); 2 Biological Chemistry & Bioactives Group, The New Zealand Institute for Plant and Food Research Limited, 120 Mount Albert Road, Mount Albert, Auckland 1025, New Zealand; emma.sherman@plantandfood.co.nz (E.S.); farhana.pinu@plantandfood.co.nz (F.R.P.)

**Keywords:** DLLME method, D-optimal design, volatile compounds, aroma, wine, GC-MS

## Abstract

**Background/Objectives**: This study reports the development of a straightforward, efficient, and cost-effective dispersive liquid–liquid microextraction (DLLME) method for the gas chromatography–mass spectrometry (GC-MS) analysis of volatile compounds present in wine. **Methods**: Four critical parameters were optimised using a D-optimal design to maximise extraction outcomes of the targeted analytes from a 10 mL sample, while minimising interference from other compounds. The analytical characteristics of the method were assessed using 36 target compounds. **Results**: The method provided satisfactory linearity (correlation coefficients > 0.990), good repeatability for both for intra- and inter-day measurements (RSD < 10.3%), and suitable recoveries of target analytes from both model (83–110%) and real matrices (80–120%). The validated method was subsequently applied to analyse the aroma profile of 30 New Zealand Pinot noir (PN) wine samples. **Conclusions**: This study contributes to the advancement of analytical techniques available to both industry and researchers to explore the complex aroma profiles of wines.

## 1. Introduction

New Zealand is increasingly recognised as one of the world’s leading producers of premium-quality wines [1]. The country’s temperate, maritime climate, long growing season, and diverse soils make it an ideal location for producing a wide range of high-quality wines, including Sauvignon blanc, Pinot noir, Chardonnay, Riesling, and Syrah, among others [2]. Aroma is one of the key elements in determining the character and quality of wine. Depending on the grape variety used, the winemaking technique employed, and the storage conditions, the wine will exhibit a complex blend of aromas and flavours, including floral, citrus, fruity, vegetal, and earthy, among many other recognised odours [3]. Additionally, the biochemical properties of wine, such as its pH, sugar, organic acid content (including tartaric, malic, and citric acids), and the presence of amino acids and peptides, contribute significantly to its sensory characteristics and the formation of aroma compounds during fermentation and ageing [4]. Some aroma compounds are known to make a positive contribution towards wine quality, while others are thought to be detrimental [5]. Although hundreds of aroma compounds are present in wine, currently available targeted analytical methods are able to identify and quantify only a small fractions of odour active compounds. Terpenes, esters, volatile phenols, norisoprenoids, alcohols, and fatty acids represent some of the important families of these compounds. Such molecules can be present only in μg/L or even ng/L concentrations yet can strongly influence the overall aroma profile of wine [6]. Gas chromatography coupled with mass spectrometry (GC–MS) is the most widely used analytical instrumentation for the analysis of aroma compounds in wine [7,8,9,10]. Depending on the wine matrix and the aroma compounds to be determined, different aroma extraction methods can be applied prior to GC–MS analysis [6].

Over the past few decades, many sampling techniques have been developed to analyse volatile compounds present in wine, with liquid–liquid extraction (LLE) methods being a commonly employed example [11,12]. The major advantage of these methods is that they can extract a broad range (high, medium, and low volatility) of compounds into the organic phase at once [11,12,13]. However, this type of method, which requires a high degree of concentration (e.g., 100-fold), is time-consuming and can lead to the degradation or loss of important aroma compounds during the evaporation step [14]. Another commonly used technique for analysing volatile compounds present in wine is solid-phase extraction (SPE) [13,15,16] which will separate target analytes according to their physical and chemical properties. One major drawback is the use of solvents in the extraction process. SPE methods generally require the use of organic solvents, which can be costly and environmentally unfriendly [17,18]. Solid-phase microextraction (SPME) is currently the most widely employed technique [19,20,21,22]. Benefits of SPME include rapid analysis time, good reproducibility, and no solvent requirement [23]. A drawback of SPME is its limited sample capacity arising from the small volume of the fibre coating, which restricts the amount of analyte that can be extracted [24]. In addition, this approach can only measure compounds that are sufficiently volatile to enter the headspace.

Recently, there has been a shift towards more economical and greener extraction techniques. Dispersive liquid–liquid microextraction (DLLME) is proposed as a more environmentally friendly sample preparation method than LLE and SPE [25]. An appropriate solvent system (comprising extraction and disperser solvents) is rapidly injected into an aqueous sample. The extraction solvent is immiscible with water while the disperser solvent is an auxiliary solvent with high solubility in both the organic and aqueous phases. The mixture of these solvents facilitates the extraction of analytes of interest present in the sample towards the organic phase. DLLME requires a significantly smaller volume of organic solvents compared to conventional LLE methods. This reduction in solvent volume leads to a decrease in the overall environmental impact associated with solvent production, usage, and disposal [26].

In 2006, Rezaee et al. [27] introduced this technique for the first time to analyse polycyclic aromatic hydrocarbons (PAHs) in water samples. Since then, this method has been applied more widely to include research areas such as pesticide analysis in water [28], analysis of neonicotinoid insecticides in grains [29], analysis of sulfonamides in milk [30], and assessing limited classes of volatiles in alcoholic beverages including wine [31,32,33,34,35,36,37]. Besides the environmental advantages that the DLLME method offers, it is also simple and fast to perform, provides good repeatability, and is cost-effective [28].

In this present study, we report the first optimisation of a DLLME method for the analysis of a wide range of aroma compounds including esters, alcohols, aldehydes, norisoprenoids, terpenes, fatty acids, sulphur compounds, and phenols in wine using GC-MS. The validated DLLME method was subsequently applied to study the aroma composition of a selection of different wines including Sauvignon blanc, Chardonnay, Pinot gris, Syrah, Pinot noir, and Merlot wines in New Zealand.

## 2. Materials and Methods

### 2.1. Reagents and Samples

HPLC-grade dichloromethane, chloroform, pentane, and hexane were supplied by SupraSolv^®^, Merck (Darmstadt, Germany). HPLC-grade acetone and acetonitrile were bought from J.T. Baker, Phillipsburg, NJ, USA, and methanol (HPLC grade) was obtained from ECP Ltd. (Auckland, New Zealand). Anhydrous sodium sulphate was obtained from ECP Ltd. (Auckland, New Zealand).

Deuterium-labelled internal standards, including *d*_3_-n-hexyl acetate, *d*_3_-2-phenylethyl acetate, *d*_3_-linalool, *d*_3_-α-terpineol, *d*_3_-3-methylbutyl acetate, *d*_12_-hexanal, and *d*_11_-hexanoic acid with over 99% purity, and *d*_11_-ethyl hexanoate, *d*_15_-ethyl octanoate, and *d*_11_-n-hexyl alcohol with over 98% purity, were purchased from CDN Isotopes (Pointe-Claire, QC, Canada). 4-Decanol (98%, Lancaster, Pelham, NH, USA), dl-3-octanol (99%, Acros Organics, Geel, Belgium), and 3,4-dimethylphenol (98%, Aldrich, Milwaukee, WI, USA) were also used.

Ultrapure water (18.2 MΩ/cm resistivity at 25 °C) was obtained from a Barnstead NANOpure^®^ Diamond™ water purification system (Thermo Fisher Scientific, Waltham, MA, USA). BOC Gases NZ Ltd. (Auckland, New Zealand) supplied the nitrogen, argon (both industrial grade), and helium (instrument grade) for the instrumental analyses.

Country red wine cask (11.5% alc/volume), a blend of different wines from Australia, New Zealand, and Argentina, was used for method development. Commercially available New Zealand wines Sauvignon blanc, Chardonnay, Pinot Gris, Syrah, Pinot noir, and Merlot were purchased from the local suppliers for method validation (for more details, refer to Table S1 in the Supplementary Materials).

### 2.2. Method Development

Four independent variables (Table 1), namely, the extraction solvent type (X_1_), extraction solvent volume (X_2_), disperser solvent type (X_3_), and disperser solvent volume (X_4_) were investigated in order to obtain optimised conditions.

The D-Optimal design was used to derive 60 conditions to explore the effects (and appropriate interactions) of these variables, which were subsequently carried out in triplicate (60 conditions as outlined in Table S2). After running the experiments, the model (outlined in Section 3.1) was fitted to the data to understand the optimal conditions for each of the aroma compounds present in wine. In the analysis, the terms were deemed to be significant if the associated *p*-value was <0.05. All non-significant terms were removed iteratively from each model until only significant effects remained. The models were then used to find the optimal combination of factors for each analyte of interest.

R software (version 4.2.3, R Core Team, Vienna, Austria) [38], an open-source statistical program, was used to create the D-optimal design and determine the best conditions, using the DoE.wrapper package Version 0.12 [39]. The MassHunter Qualitative and Quantitative analysis software (version 10.0, Agilent, Santa Clara, CA, USA) was used in the data analysis stage to integrate the peak areas of the 36 target aroma compounds and to calculate the respective peak area ratios (Table S3). Microsoft Excel (Microsoft Office 2016) was further used to manage the data.

### 2.3. Instrumentation and Conditions

An Agilent 6890N GC system coupled with mass selective detector 5973 from Agilent (Santa Clara, CA, USA) was used for the analysis. Samples were placed on a pre-cooled G261A sample tray (9 °C) and injected using a 7683B automatic liquid sampler. Splitless mode was used to inject a 1 µL sample volume with a 250 °C front inlet temperature and 16.9 psi pressure. The carrier gas (He) was set to a flow rate of 1.2 mL/min and delivered on to an HP-INNOWax capillary column (60 m, 0.250 mm ID, 0.25 µm film thickness) for analyte separation. The oven temperature program used in this method was as follows: the initial oven temperature of 50 °C was held for 5 min, then ramped to 60 °C at a rate of 1 °C/min, and again ramped to 250 °C at a rate of 10 °C/min, where it was held for 25 min. The oven was then cooled down to 50 °C at a rate of 50 °C/min and held for 2 min, giving a total run time of 65 min. The transfer line temperature was set to 250 °C and the ion source to 230 °C while electron impact mode in the ion source was set to 70 eV. A single-quadrupole mass spectrometer was used as the detector and the temperature was set at 150 °C. The analysis was performed in scan mode for the identification and quantification of volatile compounds (35–350 *m*/*z*).

### 2.4. DLLME Procedure

An aliquot of wine (10 mL spiked with the mixture of internal standards) was placed into a 15 mL centrifuge tube. A freshly prepared extraction mixture comprising chloroform (2 mL) as the extraction solvent and acetonitrile (1 mL) as the disperser solvent were quickly injected into the centrifuge tube using a micro syringe. The mixture was gently shaken for one minute and then centrifuged (1677 × *g*, 5 min). The organic phase was isolated using a syringe, followed by drying over anhydrous sodium sulphate. Subsequently, the dried organic phase was concentrated under a gentle stream of N_2_ to a final volume of ~200 µL and injected into the GC-MS for analysis.

### 2.5. Method Validation

#### 2.5.1. Calibration Curves

Calibration curves were prepared by spiking model wine (13.5% *v*/*v* ethanol, 5 g/L tartaric acid, pH adjusted to 3.5) with increasing concentrations of aroma compounds and constant concentrations of each of the associated internal standards, to give six-point calibration curves for each compound, in duplicate (Table S4). The spiked samples were subjected to the DLLME method outlined in Section 2.4.

#### 2.5.2. Repeatability and Recovery

The repeatability of the method was evaluated by spiking model wine samples with the 36 targeted compounds at three different concentrations and performing six DLLME extractions of each of these spiked wines. This was performed on one day to assess intra-day repeatability and then repeated over two successive days to assess inter-day repeatability.

Model wine samples were spiked with known concentrations of the analytes at three different levels (Table S4, Supplementary Materials), all falling within the range of the calibration curves. The accuracy of the method was evaluated by calculating the recovery through comparison of the measured analyte concentrations with the spiked concentrations.

#### 2.5.3. The Limits of Detection (LOD) and Limits of Quantification (LOQ)

The formulae used to calculate the LOD and LOQ were LOD = 3.3 σ/S and LOQ = 10 σ/S. In these formulae, σ is the residual standard deviation of the regression line, and S is the slope of the calibration curve [40].

#### 2.5.4. Matrix Effects

Eighteen wines from six different varieties namely, Sauvignon blanc, Chardonnay, Pinot gris, Syrah, Pinot noir, and Merlot were selected for this study (refer to Table S1 in the Supplementary Materials for details of these wines). Samples of each were spiked with known concentrations of 36 aroma compounds from 0.16 μg/L to 340 mg/L pertinent to each analyte (Table S4, Supplementary Materials). The original wines and the spiked samples were extracted in triplicate following the procedure described in Section 2.4. The recovery of the analytes from these matrices was then assessed.

## 3. Results

### 3.1. Method Optimisation

The efficiency of the DLLME method is affected by various factors, and since these factors can be interrelated, a multivariate approach was deemed to be appropriate for optimising the method herein. The most influential factors, including the type and volumes of extraction and disperser solvents, were evaluated.

The extraction efficiencies of five commonly reported extraction solvents in the literature related to the DLLME method were selected to be included in this study [41]. These solvents were chloroform, dichloromethane, hexane, pentane, and a mixture of chloroform/pentane (2:1). The following properties were considered during the selection of extraction solvents: aqueous immiscibility but miscibility with the disperser solvent, high extraction efficiency towards analytes of interest, and good chromatographic behaviour.

The next step was the selection of suitable disperser solvent candidates for investigation. To form a distinct cloudy solution, the disperser solvent needs to be miscible with both the extraction solvent and the aqueous phase. This step enhances the extraction efficiency by increasing the surface contact between the aqueous phase and the organic phase [41]. Accounting for this key requirement, acetone, acetonitrile, and methanol were evaluated as disperser solvents in this study [35,42,43].

The extraction efficiencies of 36 aroma compounds representative of typical wine aroma, including esters, alcohols, aldehydes, norisoprenoids, terpenes, fatty acids, and phenols were selected for comparing the experimental conditions (Table 2).

A full factorial experimental design (based on the four variables and levels outlined in Table 1) would have given rise to 225 combinations. In order to select and investigate a more manageable number of experiments, a D-optimal design was employed in order to appropriately model all of the desired factors (and interactions) [44]. This approach significantly reduces the number of experiments required for the optimisation of parameters, while still giving comparable precision to a full factorial design. As such, a D-optimal experimental design was devised to select 60 different combinations of the factors of interest (refer to Table S2 in the Supplementary Materials for details of these) which could then be used to find the optimal combination of levels to maximise extraction efficiency and ability across the analytes of interest. Combinations involving pentane as the extracting solvent did not yield a cloudy solution. Instead, a thin organic layer was retained on top of the wine sample. This likely results from the higher volatility of pentane compared to the other solvents [45]. However, other combinations formed distinct cloudy solutions, indicating their ability to disperse easily through the aqueous phase and achieve greater extraction efficiency than pentane. Combinations such as chloroform–acetone, chloroform–acetonitrile, DCM–acetone, and DCM–acetonitrile not only gave the desired cloudy mixture, but also exhibited clear separation of the organic layer. To determine the most important factors for extraction efficiency, the relative peak areas of the 36 compounds of interest were determined using the Agilent MassHunter software (version 10.0, Agilent) and were further evaluated using R software (version 4.2.3). Three of the 36 target compounds (*β*-citronellol, *trans*-2-hexen-1-ol, and geraniol) were not detected in the wine extracted using any of the 60 solvent combinations.

**Table 2:** Validation parameters for the 36 target compounds in spiked model wine.

Compound Name	Linearity (r^2^)	Linear Range (μg/L)	LOD (μg/L)	LOQ (μg/L)	Olfactory Perception Threshold (µg/L)
*Alcohols and aldehydes*					
1-butanol	0.996	219–13,134	63.35	191.97	150,000 [46]
benzaldehyde	0.991	1–60	0.29	0.87	2000 [46]
benzyl alcohol	0.995	40–3036	11.64	38.15	200,000 [46]
isoamyl alcohol	0.992	3382–338,205	1216.22	3685.54	30,000 [46]
isobutanol	0.994	1557–155,797	439.86	1332.92	40,000 [47]
methionol	0.994	76–4554	23.8	72.12	1000 [46]
phenylethyl alcohol	0.992	1873–112,363	391.22	1185.52	14,000 [46]
*C6 compounds*					
1-hexanol	0.993	261–26,107	79.73	241.63	8000 [46]
*cis*-2-hexen-1-ol	0.991	3–346	0.82	2.49	-
*cis*-3-hexen-1-ol	0.996	31–3144	10.15	30.75	400 [46]
hexanal	0.997	10–1035	3.29	9.97	-
*trans*-2-hexen-1-ol	0.9915	2.5–251.5	1.12	3.4	-
*trans*-2-hexenal	0.991	4–392	1.17	3.55	-
*trans*-3-hexen-1-ol	0.992	8–844	1.77	5.35	1000 [25]
*Esters*					
ethyl decanoate	0.998	4–466	1.51	4.57	200 [46]
ethyl hexanoate	0.997	15–1540	3.77	11.41	14 [46]
ethyl octanoate	0.998	21–2135	5.61	17.01	5 [46]
ethyl phenyl acetate	0.995	2–242	0.77	2.32	250 [46]
hexyl acetate	0.997	16–1628	4.46	13.52	700 [22]
isoamyl acetate	0.996	62–6240	19.76	59.88	30 [46]
*β*-phenylethyl acetate	0.992	5–486	1.61	4.87	250 [46]
*Fatty acids*					
decanoic acid ^a^	0.997	2.6–106	0.81	2.44	1000 [46]
hexanoic acid ^a^	0.992	3–120	0.86	2.61	420 [46]
isobutyric acid ^a^	0.995	0.4 –15	0.09	0.27	2300 [46]
isovaleric acid ^a^	0.998	1.8–71	0.37	1.12	33 [46]
octanoic acid ^a^	0.994	1 –43	0.35	1.07	500 [46]
*C13-Norisoprenoids/Terpenes*					
geraniol	0.994	0.6–65	0.22	0.64	20 [46]
linalool	0.992	0.3–32	0.1	0.29	25 [46]
*α*-ionone	0.990	0.2–19	0.05	0.16	2.6 [47]
nerol	0.998	0.6–64	0.21	0.62	400 [48]
*α*-terpineol	0.992	0.8–77	0.18	0.54	250 [46]
*β*-citronellol	0.996	0.4–43	0.14	0.43	100 [46]
*β*-damascenone	0.991	0.2–16	0.06	0.18	0.05 [46]
*β*-ionone	0.990	0.2–25	0.07	0.23	0.09 [46]
*Volatile Phenols*					
4-ethyl guaiacol	0.998	3–316	0.88	2.66	110 [49]
4-ethyl phenol	0.995	5–524	1.57	4.75	600 [49]

^a^ The unit used to measure the concentrations of these compounds is mg/L.

As the preliminary step of the statistical analysis, a separate multivariate regression model was created for each analyte of interest. Initially, a linear model with all of the proposed main effects and interactions was fit to the data for each analyte, according to Equation (1):

Y = b_0_ + b_1_X_1_ + b_2_X_2_ + b_22_X_22_ + b_3_X_3_ + b_4_X_4_ + b_44_X_42_ + b_12_X_1_X_2_ + b_34_X_3_X_4_ + b_13_X_1_X_3_ (1)

where Y is the peak area of each aroma compound measured in wine and where X_i_ (i = 1–4) are the independent variables, and b_j_ (j = 0–4) are the coefficient values obtained through multiple linear (b_1_, b_2_, b_3_ and b_4_), quadratic (for the quantitative variables, b_22_ and b_44_) regression and the most-relevant two-factor interactions (b_12_, b_13_ and b_34_). Following the generation of the full linear regression model, a backward elimination strategy was used to sequentially remove non-significant (*p*-value ≥ 0.05) terms. As an example, for ethyl octanoate, all quadratic regression terms, two interaction terms, and one main effect linear term were greater than 0.05, and therefore, those terms were discarded from the model during this process. The final modified equation (Equation (2)) for the ethyl octanoate is as shown:

Y1 = b_0_ + b_1_X_1_ + b_2_X_2_ + b_3_X_3_ + b_12_X_1_X_2_ (2)

Eg: Ethyl octanoate = 58,296 + 36,300X_ExtSolTypeChloroform_Pentane_ + 3872X_ExtSolTypeDCM_ + 13,362X_ExtSolTypeHexane_ + 173,542X_ExtSolTypePentane_ + 36X_ExtSolventVolume_ – 29,513X_DisperseTypeAcetonitrile_ – 20,206X_DisperseTypeMethanol_ − 38X_ExtSolTypeChloroform_Pentane:ExtSolVolume_ − 18X_ExtSolTypeDCM:ExtSolVolume_ + 9X_ExtSolTypeHexane:ExtSolVolume_ − 126X_ExtSolType_Pentane:ExtSolVolume_

**Figure 1:**
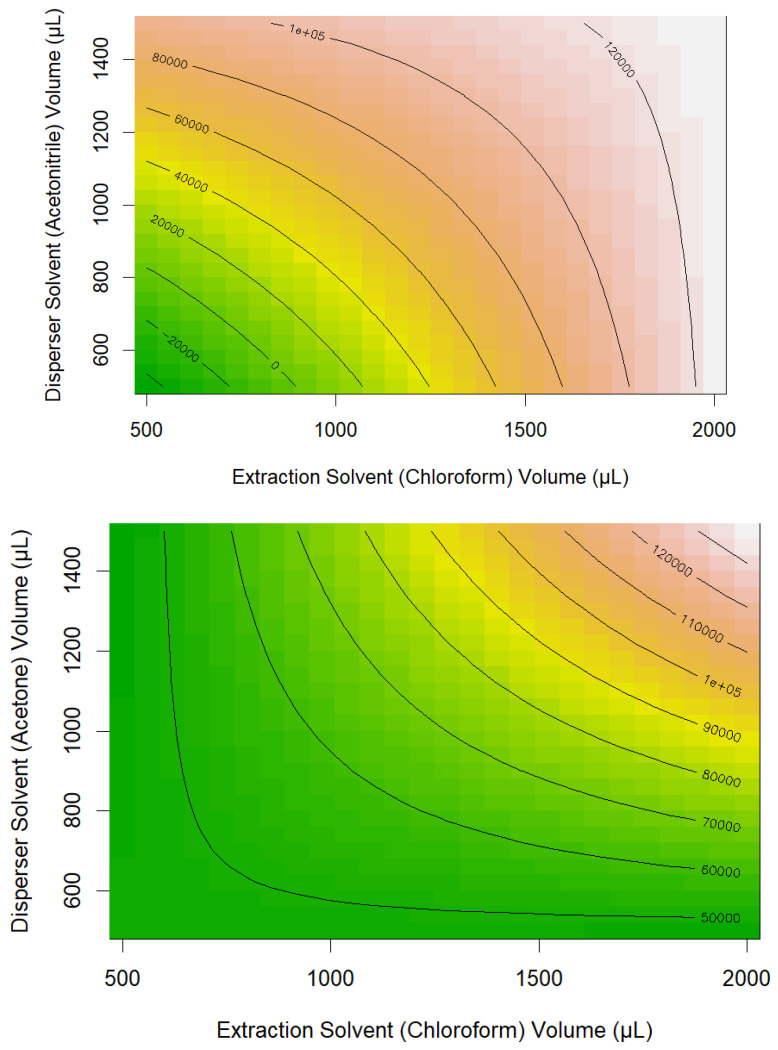

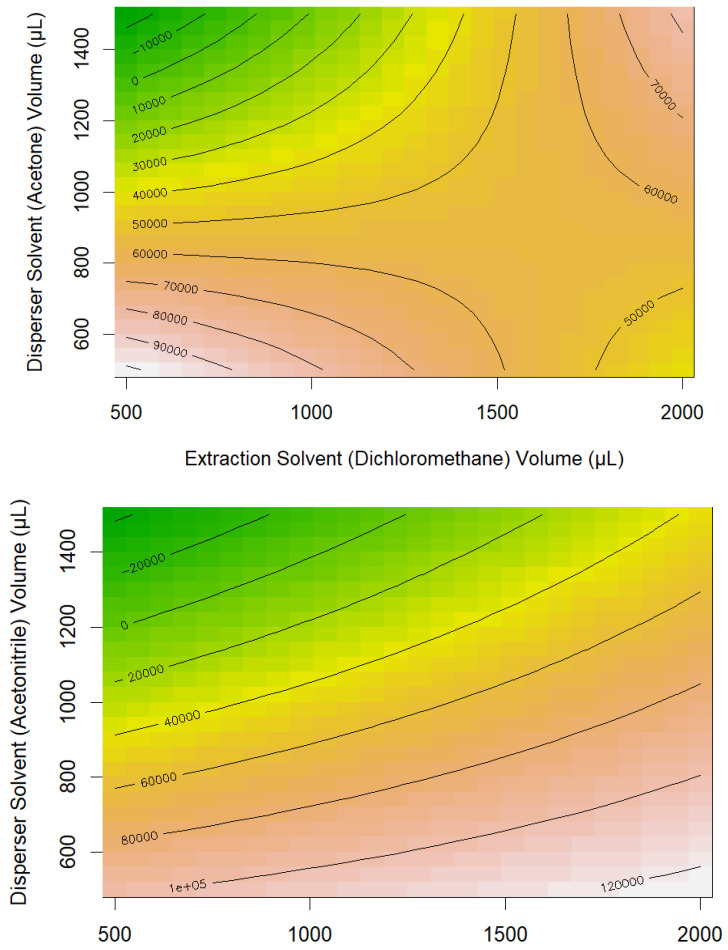
Examples of variation in ethyl octanoate extraction efficiency (measured as chromatogram peak area) using different extraction solvents (dichloromethane or chloroform), disperser solvents (acetone or acetonitrile), and solvent volumes.

In this case, the extraction solvent type, extraction solvent volume, disperser solvent type, and the two-factor interaction between extraction solvent type and volume were four significant terms in the regression model. The extraction solvent type and the two-factor interaction between extraction solvent volume and extraction solvent type gave the lowest significant *p*-values, indicating that these two factors have the greatest evidence of their influence on ethyl octanoate extraction efficiency. We found that for the regression models of all of the studied analytes, generally, the quadratic terms for the quantitative variables (i.e., the solvent volumes) were not significant, and two-factor interactions and linear main effects were the parameters that most often influenced the extraction efficiency (Figure 1). Following the establishment of a regression model for each analyte, the developed models were used to interpolate the peak areas of each aroma compound for all the possible combinations of levels of the parameters (225 in total). Some compounds were predicted to have negative values for their peak area and these negative predictions were replaced with “0”.

**Figure 2:**
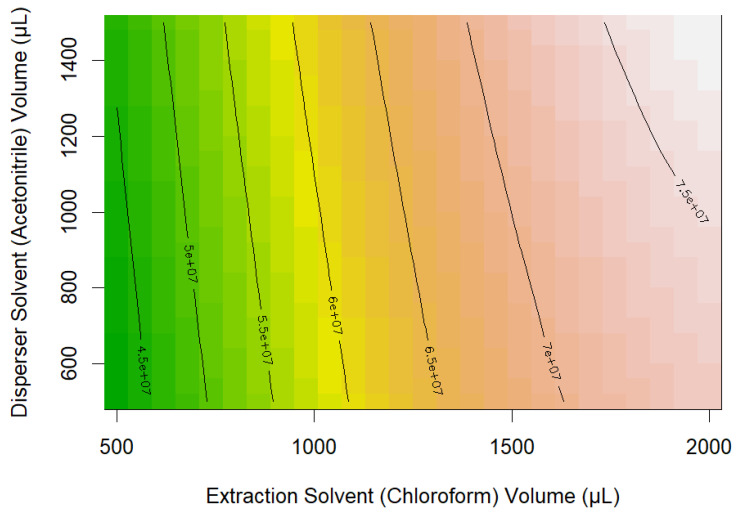
Variation in total chromatogram peak area as a function of chloroform extraction solvent volume and acetonitrile disperser solvent volume.

The 10 best conditions were selected by two approaches. The first approach was to calculate the total peak area for every predicted combination by summing all of the peak areas of the 36 compounds of interest. The top combinations were selected based on this highest total peak area value (Table S5, Supplementary Materials). Combinations with chloroform as the extracting solvent and acetonitrile as the disperser solvent can be seen to be the highest-performing under this ranking scheme. Chloroform (2 mL) as the extraction solvent and acetonitrile (1.5 mL) as the disperser solvent gave the highest predicted total peak area (Figure 2).

The second approach was to identify the top three sets of conditions that gave the highest extraction efficiencies for each aroma compound (36 compounds) and note them as best performers for that analyte of interest. By performing this for all of the analytes of interest and summing the number of times a particular combination of conditions appeared, the top 10 were selected. Table S6 (Supplementary Materials) shows these best 10 performers according to the total number of compounds with a top-three extraction efficiency under these conditions. It was noted that the widest range of aroma compounds classes, including alcohols, terpenes, C6 compounds, esters, fatty acids, and phenols, were extracted well by a combination of chloroform and acetonitrile.

**Figure 3:**
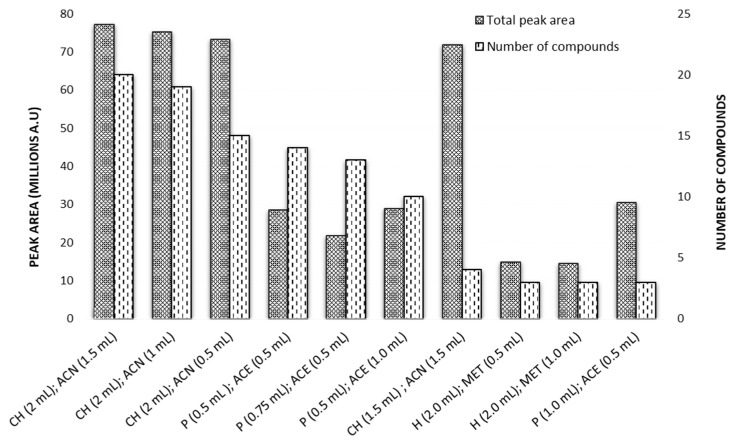
Top ten DLLME solvent combination performers according to the total number of compounds with a top-3 extraction efficiency. Extraction solvent type (v, mL); disperser solvent type (v, mL). CH, chloroform; P, pentane; H, hexane; ACN, acetonitrile; ACE, acetone; MET, methanol.

From the results in Tables S5 and S6, it was clear that the three best predicted responses using the developed models were with chloroform as the extraction solvent and acetonitrile as disperser solvent type (Figure 3). The extracts obtained from these combinations also showed good chromatographic behaviour. The calculated relative standard deviations (RSDs) for the four samples extracted in the laboratory (i.e., run as combinations present in the D-optimal design) with the chloroform and acetonitrile were all less than 10%. The top three combinations were found to be the same under both ranking criteria. These combinations all have chloroform (2 mL) as the extracting solvent and acetonitrile (either 0.5, 1 or 1.5 mL) as the disperser solvent. Little separated the top two (1 mL and 1.5 mL acetonitrile), given both perform well extracting a diverse selection of compounds (including alcohols, terpenes, C6 compounds, esters, fatty acids, and phenols). Therefore, in the interest of limiting solvent waste, toxicity, and method cost, acetonitrile 1.0 mL was deemed preferable over its 1.5 mL disperser solvent counterpart.

As such, a mixture of chloroform (2.0 mL) as the extracting solvent and acetonitrile (1.0 mL) as the disperser solvent was selected as the optimal extraction combination for this DLLME method.

### 3.2. Method Validation

#### 3.2.1. Linearity, LOD, and LOQ

Using the optimised DLLME method identified in Section 3.1, calibration curves were prepared for seven esters, seven alcohols, seven C6 compounds, three norisoprenoids, five terpenes, five fatty acids, and two phenols. Model wine (13.5% v/v ethanol, 5 g/L tartaric acid, pH adjusted to 3.5) was spiked with 36 compounds at six different concentration levels and subsequently analysed. The GC-MS response displayed good linearity for all aroma compounds within the test range, as evidenced by linear regression coefficients (r^2^) exceeding 0.99 (Table 2). Notably, the C13-norisoprenoids exhibited the lowest r^2^ value in this study due to their high boiling point, which has been reported to make their extraction challenging, especially at very low concentrations [33]. The linear range, correlation coefficient, LOD, and LOQ for each compound are reported in Table 2.

#### 3.2.2. Method Repeatability and Recovery

The repeatability of the method was tested by analysing six model wine samples spiked at three different levels (low, medium, and high). These concentration levels are presented in Table S4. Table 3 reports the accuracy and precision outcomes of the proposed approach for examining each of the 36 volatile compounds in wine. The precision (standard deviation) of all compounds ranged from 0.1 to 10.3%, which suggests that the proposed approach for analysing the compounds falls within the acceptance range established by SANTE/SANCO and EU directive 2002/657/EC [50]. The accuracy of the method was assessed by conducting recovery tests for each of the volatile compounds studied at three distinct concentrations from model wine. All of the recovery percentages for aroma components, which range from 83 to 110% and are shown in Table 3, were within the acceptable range [50].

#### 3.2.3. Evaluation of Possible Matrix Effects of the DLLME Method

To assess possible matrix effects, the optimised method was applied in triplicate to samples of Sauvignon blanc, Chardonnay, Pinot Gris, Syrah, Pinot noir, and Merlot wines (details of these wines can be found in Table S1). These wines were spiked with the 36 target compounds at the same three levels as the model wine (Table S4). The formula of R = (Co − Cb)/Cs × 100, where R is the recovery value, Co is the calculated concentration in the spiked matrix, Cb is the calculated concentration in the non-spiked matrix, and Cs is the spiked concentration in the matrix, was used [51]. Satisfactory recoveries for all the compounds were acquired from all six wine varieties investigated; the recoveries were in the range of 80–120%, and all relative standard deviations were below 10% (see Table S7 for these values).

### 3.3. Method Application

The newly validated DLLME method was subsequently applied to measure the concentrations of volatile compounds present in 30 New Zealand 2020 vintage Pinot noir wines (Table S8 in Supplementary Materials) produced in three different regions (Marlborough, Central Otago, and Martinborough). These wines were purchased from commercial retail shops across New Zealand. The extractions were performed in triplicate, and the concentration ranges for the 10 wines from each region are displayed in Table 4.

Among the esters, ethyl hexanoate, ethyl octanoate, hexyl acetate, and isoamyl acetate were found to be, on average, more abundant in the Marlborough wines, while *β*-phenylethyl acetate was, on average, in highest concentration in the Central Otago wines at 13.42 µg/L. Martinborough had the highest concentration of *β*-damascenone among the C13 norisoprenoids at 8.91 µg/L. This value is in good agreement with the estimate of Pineau et al. (2007), who reported a range of 2 to 7 mg/L for red wine [52]. Geraniol was the terpene present in the highest concentration across all three regions, ranging from 14.1 to 16.4 µg/L. The geraniol concentration in the late-harvest Pinot noir wines from 2013 was found to be 24.2 µg/L, as reported by Fang and Qian (2006). This value is greater than the one observed in this study and is in agreement with the early-stage concentration of 14.7 µg/L [53]. Bergler et al. (2020) analysed terpene concentrations in Chardonnay and Ugni Blanc wines using the DLLME method, reporting geraniol concentrations between 6 and 37 µg/L and linalool concentrations from 9 to 44 µg/L [34]. In this study, geraniol concentrations were consistent with those reported by Bergler et al., while the linalool concentrations were lower than the reported range. Among the fatty acids, hexanoic acid was the most abundant in all three regions, with concentrations ranging from 4763.6 to 8943.3 µg/L. The concentration of hexanoic acid in 2010 Premium wine (712 µg/L) and 2009 Estate wine (1217 µg/L) reported by Rutan et al. (2014) were lower than the concentrations observed in this study [9]. This could be attributed to variations in extraction methods or differences among wine varieties [54].

The concentration of isoamyl alcohol was the highest among all the regions, varying from 122,862 to 269,955 µg/L. Rutan et al. (2014) reported a lower range of 104,295 to 150,538 µg/L for isoamyl alcohol in Pinot noir wines from Central Otago [9]. Among the phenols, 4-ethylphenol was found to be the highest in the wines produced in Central Otago with a concentration of 521.9 µg/L. In comparison, Rutan et al. (2014) reported a range of 421–727 µg/L for 4-ethylphenols in Pinot noir wines [9]. According to the literature, C6-alcohols such as 1-hexanol, *cis*-3-hexenol, and *trans*-3-hexenol can accumulate to high concentrations in Pinot noir grapes and are often associated with “cut-grass” aromas [55]. As per the findings of this investigation, 1-hexanol was the most abundant among the C6 compounds in all three regions, with concentrations ranging from 1563.9 to 3643.1 µg/L.

## 4. Conclusions

This study has reported the optimisation of a straightforward, efficient, and cost-effective DLLME method for the GC-MS analysis of volatile aroma compounds in wine. A D-optimal design was utilised to model extraction condition responses in order to choose the best experimental parameters. The developed method showed good linearity, recovery, and repeatability, and was subsequently applied to the analyses of 36 aroma compounds in 30 New Zealand Pinot noir wines. This study contributes to the advancement of analytical techniques to support the wine industry, providing a valuable tool for exploring the complex aroma profiles of wines.

## Figures and Tables

**Table 1:** Extraction solvents, disperser solvents, extraction solvent volumes, and disperse solvent volumes investigated.

Extraction Solvent Type (X_1_)	Extraction Solvent Volume (µL) (X_2_)	Disperser Solvent Type (X_3_)	Disperser Solvent Volume (µL) (X_4_)
Chloroform (CH)	500	Acetone	500
Dichloromethane (DCM)	750	Acetonitrile	1000
Hexane (H)	1000	Methanol	1500
Pentane (P)	1500		
CH:P (2:1)	2000		

**Table 3:** Recovery and precision parameters in spiked model wine at three different concentrations using the optimised DLLME procedure.

	Intra-Day Precision (RSD%)			Inter-Day Precision (RSD%)			Recovery (%)		
Aroma Compound	Low	Medium	High	Low	Medium	High	Low	Medium	High
*Alcohols and Aldehydes*									
1-butanol	1.3	1.0	1.0	10.3	6.2	7.8	83.1	94.6	102.7
benzaldehyde	1.8	0.5	3.0	8.0	2.7	5.3	86.5	84.2	104.1
benzyl alcohol	1.5	0.4	3.7	3.4	3.7	7.4	84.7	98.4	84.9
isoamyl alcohol	0.5	0.1	0.5	3.1	2.9	5.6	89.1	97.5	106.0
isobutanol	1.1	1.7	1.0	2.5	2.7	2.1	85.8	96.6	98.2
methionol	0.8	1.9	2.4	4.2	2.6	7.3	88.2	83.4	105.4
phenylethyl alcohol	0.6	0.7	1.6	3.9	2.3	3.1	97.1	102.7	107.2
*C6 Compounds*									
1-hexanol	9.0	2.5	0.7	2.1	4.2	4.0	88.8	96.0	109.4
*cis*-2-hexen-1-ol	1.3	2.9	6.6	1.2	4.9	5.7	99.3	88.9	105.6
*cis*-3-hexen-1-ol	5.3	1.3	0.4	7.5	4.9	0.6	96.1	101.7	87.4
hexanal	6.5	6.9	5.3	7.2	3.0	6.7	97.2	84.0	99.2
*trans*-2-hexen-1-ol	0.5	0.1	0.7	3.3	0.9	2.9	91.9	94.9	91.6
*trans*-2-hexenal	0.5	0.1	0.7	1.0	1.5	1.2	83.1	94.4	95.2
*trans*-3-hexen-1-ol	5.3	3.3	0.9	6.9	4.6	3.7	95.9	99.7	98.0
*Esters*									
ethyl decanoate	1.8	2.7	5.5	8.3	8.3	4.3	92.0	102.7	85.5
ethyl hexanoate	1.3	1.6	5.9	3.9	1.9	6.8	89.6	100.6	103.3
ethyl octanoate	1.8	0.2	1.0	1.6	1.8	0.6	103.9	83.4	95.2
ethyl phenyl acetate	2.1	0.3	1.3	1.3	4.6	7.8	94.8	95.1	99.7
hexyl acetate	1.9	0.5	4.2	1.0	4.5	4.9	92.1	101.8	97.2
isoamyl acetate	1.8	1.1	4.6	3.2	7.3	1.7	103.5	85.7	100.1
*β*-phenylethyl acetate	1.1	0.4	2.0	1.4	7.7	5.4	96.2	96.3	87.2
*Fatty acids*									
decanoic acid	3.0	1.7	1.6	7.7	8.8	2.4	83.5	87.2	104.8
hexanoic acid	0.8	0.6	1.9	8.7	3.8	2.8	85.6	89.0	108.1
isobutyric acid	3.6	1.6	2.0	5.7	3.1	7.3	91.7	95.9	91.3
isovaleric acid	0.8	0.2	0.7	7.4	7.4	3.2	90.0	102.7	94.3
octanoic acid	2.7	1.5	2.4	7.6	8.6	2.1	89.1	96.5	85.8
*C13-Norisoprenoids/Terpenes*									
geraniol	4.9	1.9	5.3	4.7	4.7	6.3	85.1	96.2	98.5
linalool	4.3	3.1	1.5	9.8	7.1	5.3	84.1	101.9	99.3
nerol	1.1	1.9	0.8	4.5	2.9	6.7	85.1	96.2	98.5
*α*-ionone	2.5	1.8	3.1	3.6	6.8	7.9	96.5	98.6	93.3
*α*-terpineol	2.0	3.9	4.1	10.0	8.4	6.0	85.8	102.6	88.6
*β*-citronellol	5.2	4.7	2.7	7.6	5.2	5.7	101.2	86.5	75.8
*β*-damascenone	3.7	6.6	3.9	4.6	4.9	4.3	91.6	85.6	91.3
*β*-ionone	4.1	3.2	1.5	6.3	9.3	3.1	91.2	88.8	88.2
*Volatile Phenols*									
4-ethyl guaiacol	2.2	0.4	0.2	5.2	1.4	1.8	91.0	84.3	83.2
4-ethyl phenol	4.9	4.8	2.1	5.0	7.0	2.0	98.9	102.6	102.3

**Table 4:** Concentration ranges of 36 aroma compounds measured in 30 New Zealand 2020 Pinot noir wines using the newly validated DLLME method. Nd: not detected.

Aroma Compound	Concentration (μg/L)					
	Central Otago		Marlborough		Martinborough	
	(N = 10)		(N = 10)		(N = 10)	
	Min	Max	Min	Max	Min	Max
*Alcohols and aldehydes*						
1-butanol	1183.0	2453.3	1208.0	2168.2	1267.9	2382.6
benzaldehyde	5.2	8.6	5.7	10.8	4.9	15.6
benzyl alcohol	942.1	1788.0	1709.1	2806.6	1550.3	2917.9
isoamyl alcohol	125,040.4	202,488.9	136,642.9	235,837.5	122,862.0	269,954.2
isobutanol	13,331.6	25,277.5	14,180.5	30,897.4	12,558.4	20,654.1
methionol	1001.2	2942.0	663.8	2443.3	514.4	1975.9
phenylethyl alcohol	47,107.6	95,503.5	54,703.6	98,159.9	49,529.3	94,846.6
*C6 compounds*						
1-hexanol	1563.9	3451.2	1690.7	3643.1	1817.5	2836.7
*cis*-2-hexen-1-ol	19.5	49.8	12.6	45.9	19.4	95.9
*cis*-3-hexen-1-ol	35.8	65.8	32.8	67.2	32.9	42.9
hexanal	410.2	715.1	305.7	507.6	520.2	880.3
*trans*-2-hexen-1-ol	Nd	Nd	Nd	Nd	Nd	Nd
*trans*-2-hexenal	271.3	333.3	292.7	352.9	215.3	364.3
*trans*-3-hexen-1-ol	11.0	39.9	11.3	19.2	11.5	20.5
*Esters*						
ethyl decanoate	127.9	284.6	107.1	199.1	102.3	438.0
ethyl hexanoate	235.3	516.6	230.3	615.6	221.4	467.7
ethyl octanoate	185.7	554.6	308.7	710.2	157.1	611.9
ethyl phenyl acetate	3.1	14.3	2.9	10.9	3.2	7.6
hexyl acetate	26.6	62.1	40.3	96.0	26.0	87.1
isoamyl acetate	116.9	296.5	154.6	469.5	89.3	261.1
*β*-phenylethyl acetate	9.2	13.4	8.9	11.5	8.8	12.9
*Fatty acids*						
decanoic acid	2877.0	3046.7	2885.3	3902.7	2706.3	4461.6
hexanoic acid	5053.0	8709.3	4763.6	8943.3	4926.7	8670.3
isobutyric acid	661.0	933.6	505.0	973.3	411.0	1057.6
isovaleric acid	1888.4	3750.1	2620.3	5920.9	2130.6	5430.0
octanoic acid	3101.0	5694.0	3740.1	6915.0	2223.3	5826.7
*C13-Norisoprenoids/Terpenes*						
geraniol	14.9	16.4	14.1	16.3	14.3	16.4
linalool	1.9	2.6	1.9	2.8	1.9	2.2
nerol	2.4	4.4	2.4	6.9	2.6	3.8
*α*-ionone	0.3	0.9	0.3	0.5	0.3	0.5
*α*-terpineol	3.7	4.4	3.3	4.5	3.7	6.2
*β*-citronellol	Nd	Nd	Nd	Nd	Nd	Nd
*β*-damascenone	1.3	3.6	2.3	4.6	2.1	8.9
*β*-ionone	0.3	0.9	0.3	0.7	0.3	1.0
*Volatile Phenols*						
4-ethyl guaiacol	3.5	5.7	3.2	7.1	3.0	7.9
4-ethyl phenol	312.7	521.9	158.9	509.4	157.9	438.4

## Data Availability

The raw data supporting the conclusions of this article will be made available by the authors on request.

## Supplementary Materials

The following supporting information can be downloaded at: https://www.mdpi.com/article/10.3390/metabo15020129/s1, Table S1: Details of the eighteen wines included in the method validation experiments. Table S2: Different experimental combinations (D-optimal experimental design) explored. Table S3: Target aroma compounds and their associated internal standards, target ions, and retention times used for GC-MS. Table S4: Concentration levels of accuracy and precision parameters. Table S5: Solvent combinations with the highest predicted total GC-MS chromatogram peak areas (36 target compounds and internal standards were included). Table S6: Best conditions according to the total number of compounds. Table S7: Recovery (%) of aroma compounds in different real wine matrices using the DLLME method. Values are reported as a range from three different wines of the same variety at three different spiked concentrations. Table S8: Details of the 30 NZ PN wines (2020 vintage) included in the method application study.
