# Supplementary material for: Development of a Dispersive Liquid–Liquid Microextraction Method for Quantification of Volatile Compounds in Wines Using Gas Chromatography–Mass Spectrometry
Source: Metabolites. 2025 Feb 13;15(2):129. doi: 10.3390/metabo15020129 (PMC11857230; doi:10.3390/metabo15020129)
Supplement: Supplementary file 1 [file metabolites-15-00129-s001.zip › metabolites-3344417-supplementary.pdf]

Supplementary Information

Development of a dispersive liquid–liquid microextraction method for quantification of volatile compounds in wines using gas chromatography–mass spectrometry

Dinesha Katugampala Appuhamilage, Rebecca E. Jelley, Emma Sherman,
Lisa I. Pilkington, Farhana Pinu, Bruno Fedrizzi

Table S1: Details of the eighteen wines included in the method validation experiments.

Wine	Vintage	Region
Villa Maria Cellar Selection Merlot	2020	Hawke’s Bay
Mount Ridge By Giesen Merlot	2020	Hawke’s Bay
Allan Scott Hawkes Bay Merlot	2020	Hawke’s Bay
Villa Maria Earth Garden Pinot Noir	2020	Marlborough
Framingham Nobody’s Hero Pinot Noir	2020	Marlborough
Lake Chalice Raptor Pinot Noir	2020	Marlborough
Theory And Practice Chardonnay	2020	Hawke’s Bay
Squawking Magpie Gravels Chardonnay	2020	Hawke’s Bay
Sacred Hill Reserve Chardonnay	2020	Hawke’s Bay
Bilancia Syrah	2020	Hawke’s Bay
Clearview Reserve Syrah	2020	Hawke’s Bay
Clearview Cape Kidnappers Syrah	2020	Hawke’s Bay
Villa Maria Pinot Gris Reserve	2020	Marlborough
Devils Staircase Pinot Gris	2020	Central Otago
Ata Rangi Lismore Pinot Gris	2020	Martinborough
Akarua Central Otago Sauvignon Blanc	2020	Central Otago
Allan Scott Marlborough Sauvignon Blanc	2021	Marlborough
Neudorf Tiritiri Sauvignon Blanc	2019	Nelson

Table S2: Different experimental combinations (D-optimal experimental design) explored.

	Extraction Solvent	Extraction Solvent Volume (μL)	Disperser Solvent	Disperser Solvent Volume (μL)
1	Chloroform/Pentane	500	Methanol	500
2	Hexane	1500	Acetonitrile	1500
3	Chloroform/Pentane	1500	Acetonitrile	1000
4	Dichloromethane	500	Methanol	1500
5	Chloroform	500	Methanol	500
6	Pentane	2000	Methanol	1000
7	Pentane	500	Methanol	1500
8	Chloroform/Pentane	2000	Methanol	500
9	Pentane	500	Acetonitrile	500
10	Hexane	500	Acetonitrile	1500
11	Hexane	2000	Methanol	500
12	Chloroform	2000	Acetone	500
13	Pentane	1000	Acetone	1500
14	Pentane	750	Methanol	500
15	Pentane	750	Acetonitrile	1500
16	Dichloromethane	2000	Methanol	1000
17	Hexane	500	Acetone	1500
18	Chloroform	1000	Acetone	1500
19	Chloroform	500	Methanol	1500
20	Pentane	2000	Acetonitrile	1500
21	Dichloromethane	500	Acetone	1000
22	Chloroform	1500	Methanol	500
23	Hexane	750	Methanol	1000
24	Chloroform	750	Acetonitrile	500
25	Chloroform	2000	Acetone	1500
26	Chloroform/Pentane	1500	Methanol	1500
27	Hexane	1500	Acetone	500
28	Dichloromethane	500	Methanol	500
29	Pentane	2000	Methanol	1500
30	Dichloromethane	500	Acetone	500
31	Hexane	500	Acetone	1000
32	Dichloromethane	2000	Acetonitrile	500
33	Dichloromethane	500	Acetonitrile	1000
34	Pentane	500	Acetone	500
35	Dichloromethane	2000	Acetonitrile	1500
36	Hexane	2000	Methanol	1500
37	Chloroform	2000	Acetonitrile	1500
38	Chloroform	500	Acetone	1000
39	Pentane	2000	Acetonitrile	500
40	Chloroform/Pentane	2000	Acetone	1000
41	Hexane	2000	Acetone	1500
42	Hexane	2000	Acetonitrile	1000
43	Chloroform/Pentane	2000	Acetonitrile	1500
44	Dichloromethane	1500	Methanol	1500

45	Chloroform	2000	Acetonitrile	1000
46	Pentane	2000	Acetone	1500
47	Hexane	500	Methanol	1500
48	Chloroform	500	Acetonitrile	1500
49	Dichloromethane	2000	Acetone	1500
50	Dichloromethane	2000	Acetone	500
51	Chloroform/Pentane	500	Methanol	1500
52	Chloroform/Pentane	500	Acetonitrile	500
53	Pentane	2000	Acetone	500
54	Chloroform/Pentane	750	Acetone	500
55	Chloroform	2000	Methanol	1000
56	Dichloromethane	1000	Acetonitrile	500
57	Chloroform/Pentane	500	Acetonitrile	1500
58	Hexane	500	Acetonitrile	500
59	Chloroform/Pentane	2000	Acetone	1500
60	Chloroform/Pentane	500	Acetone	1000

Table S3 Target aroma compounds and their associated internal standards, target ions and retention times used for GC-MS.

**Compound name**	**Internal standard**	**Retention time [min]**	**Target ions [*m*/*z*]***
Alcohols and aldehydes			
1-butanol	DL-3-octanol	17.6	**56**, 31, 41
benzaldehyde	DL-3-octanol	27.0	77, **106**, 105
benzyl alcohol	DL-3-octanol	31.4	**107,** 79, 108
isoamyl alcohol	DL-3-octanol	20.3	55, 42, **70**
isobutanol	DL-3-octanol	16.5	43, **41**, 74
methionol	DL-3-octanol	29.4	**106**, 61, 58
phenylethyl alcohol	DL-3-octanol	31.8	**91**, 92, 122
C6 compounds			
1-hexanol	d_{11}-n-hexyl alcohol	23.8	**56**, 43, 69
cis-2-hexen-1-ol	d_{11}-n-hexyl alcohol	25.0	**57**, 67, 82
cis-3-hexen-1-ol	d_{11}-n-hexyl alcohol	24.5	**67**, 41, 82
hexanal	d_{12}-hexanal	20.7	**44**, 56, 57, 72
trans-2-hexen-1-ol	d_{11}-n-hexyl alcohol	31.8	**57,** 41, 82
trans-2-hexenal	d_{12}-hexanal	20.7	**41**, 83, 69
trans-3-hexen-1-ol	d_{11}-n-hexyl alcohol	24.1	41, 67, **82**
Esters			
ethyl decanoate	d_{15}-ethyl octanoate	28.3	**88**, 101, 155
ethyl hexanoate	d_{11}-ethyl hexanoate	21.1	**88**, 99, 101
ethyl octanoate	d_{15}-ethyl octanoate	25.4	88, **101**,127
ethyl phenyl acetate	d_3-2-phenylethyl acetate	30.0	**91**,106, 164
hexyl acetate	d_3-n-hexyl acetate-	22.1	56, **61**, 84
isoamyl acetate	d_3-3-methylbutyl acetate	17.4	70, 55, **87**
β-phenylethyl acetate	d_3-2-phenylethyl acetate	30.7	104, **43**, 91
Fatty acids			
decanoic acid	4-decanol	35.2	**60**, 73, 129
hexanoic acid	d_{11}-hexanoic acid	30.9	**60**, 73, 87
isobutyric acid	d_{11}-hexanoic acid	27.4	**43**, 73, 88
isovaleric acid	d_{11}-hexanoic acid	28.7	43**, 60**, 87
octanoic acid	DL-3-octanol	33.1	**60**, 73, 101
C13Norisoprenoids/Terpenes			
geraniol	d_3-linalool	30.5	**69**, 41, 93,123
linalool	d_3-linalool	27.0	71, **93**, 121
nerol	d_3-linalool	30.8	**69**, 41, 93, 121
α-ionone	d_3-linalool	31.2	**121**, 136, 192
α-terpineol	d_3-α-terpineol	29.2	**59**, 93, 121
β-citronellol	d_3-linalool	29.5	41,**69**,82,123
β-damascenone	d_3-linalool	30.8	69, **121**, 190
β-ionone	d_3-linalool	32.1	**177**, 178, 192
Phenols			

4-ethyl guaiacol	3,4-dimethyl phenol	33.1	**137**, 152, 39
4-ethyl phenol	3,4-dimethyl phenol	34.0	**107**, 122, 77

* Quantifier ion indicated in bold

Table S4: Concentration levels of Accuracy and precision parameters.

Aroma compound	Concentration (µg/L)		
Alcohols/Aldehydes	Low	Medium	High
1-butanol	218.9	4378	13134
benzaldehyde	1	19.96	59.88
benzyl alcohol	39.11	782.26	2346.77
isoamyl alcohol	3382.11	782.26	338206
isobutanol	1557.97	38949.3	116848
methionol	75.91	1518.22	4554.65
phenylethyl alcohol	1872.72	37454.5	112363
C6 Compounds			
1-hexanol	261.07	6526.8	26107.2
cis-2-hexen-1-ol	3.46	86.53	346.1
cis-3-hexen-1-ol	31.44	786.04	3144.15
hexanal	10.35	258.72	1034.88
trans-2-hexen-1-ol	2.52	62.9	251.59
trans-2-hexenal	3.92	97.96	391.84
trans-3-hexen-1-ol	8.44	210.97	843.86
Esters			
ethyl decanoate	4.66	116.6	466.41
ethyl hexanoate	15.4	385.08	1540.32
ethyl octanoate	21.34	533.41	2133.65
ethyl phenylacetate	2.42	60.51	242.04
hexyl acetate	16.28	406.93	1627.72
isoamyl acetate	62.4	1560	6240
β-phenylethyl acetate	4.86	121.45	485.81
Fatty acids			
decanoic acid	2.65	53.04	106.09
hexanoic acid	3.01	60.15	120.3
isobutyric acid	0.35	7.01	14.03
isovaleric acid	1.67	33.49	66.97
octanoic acid	1.07	21.39	42.78
C13-Norisoprenoids/Terpenes			
geraniol	0.65	16.3	65.18
linalool	0.32	7.89	31.54

nerol	0.64	16.1	64.41
α-ionone	0.19	4.69	18.75
α-terpineol	0.77	19.16	76.63
β-citronellol	0.43	10.8	43.2
β-damascenone	0.16	4.04	16.15
β-ionone	0.25	6.25	25
Volatile Phenols			
4-ethyl guaiacol	3.16	78.91	315.64
4-ethyl phenol	5.24	130.96	523.84
Internal standards			
d_{11}-Ethyl hexanoate	1178	1178	1178
d_{15}-Ethyl octanoate	1090	1090	1090
d_3-3-Methylbutyl acetate	1194	1194	1194
d_3-n-Hexyl acetate	659	659	659
d_3-2-Phenylethyl acetate	292	292	292
d_3-Linalool	76	76	76
d_3-α-Terpineol	75	75	75
d_{12}-hexanal	67	67	67
d_{11}-n-hexyl alcohol	1189	1189	1189
DL-3-Octanol	398	398	398
4-Decanol	456	456	456
3,4-Dimethylphenol	156	156	156
d_{11}-Hexanoic acid	8253	8253	8253

Table S5: Solvent combinations with the highest predicted total GC-MS chromatogram peak areas (36 target compounds and internal standards were included).

**Rank**	**Extraction solvent**		**Disperser solvent**		**Peak area (A.U)**
	Solvent	Volume (mL)	Solvent	Volume (mL)	
1	Chloroform	2.0	Acetonitrile	1.5	77101919.03
2	Chloroform	2.0	Acetonitrile	1.0	75179091.35
3	Chloroform	2.0	Acetonitrile	0.5	73262862.31
4	Chloroform	1.5	Acetonitrile	1.5	71922990.88
5	Chloroform	1.5	Acetonitrile	1.0	69983717.18
6	Chloroform	1.5	Acetonitrile	0.5	68166221.46
7	Chloroform	1.0	Acetonitrile	1.5	61469369.13
8	Chloroform	2.0	Acetone	1.5	61315472.54
9	Chloroform	2.0	Acetone	1.0	60900520.88
10	Chloroform	2.0	Acetone	0.5	60492167.86

Table S6: Best conditions according to the total number* of compounds.

Rank	Extraction solvent		Disperse solvent		Compound Groups	Number of Compounds*
	Solvent	volume (mL)	Solvent	volume (mL)		
1	Chloroform	2.0	Acetonitrile	1.5	Alcohols, Terpenes, C6 compounds, Esters, Fatty acids and phenols	20
2	Chloroform	2.0	Acetonitrile	1.0	Alcohols, Terpenes, C6 compounds, Esters, Fatty acids and Phenols	19
3	Chloroform	2.0	Acetonitrile	0.5	Terpenes, Alcohols and Phenols	15
4	Pentane	0.5	Acetone	0.5	Esters, Aldehydes, Terpenes, and Alcohols	14
5	Pentane	0.75	Acetone	0.5	Esters, Terpenes, Alcohols and Phenols	13
6	Pentane	0.5	Acetone	1.0	Esters, Terpenes and Alcohols	10
7	Chloroform	1.5	Acetonitrile	1.5	Fatty acids, alcohols and Terpenes	4
8	Hexane	2.0	Methanol	0.5	Esters and Terpenes	3
9	Hexane	2.0	Methanol	1	Esters and Terpenes	3
10	Pentane	1.0	Acetone	0.5	Alcohols, Esters and Terpenes	3

*Total number of compounds was defined as the frequency a solvent combination was ranked in the top three by predicted GC-MS peak area for one of the 36 target aroma compounds or internal standards.

Table S7: Recovery (%) of aroma compounds in different real wine matrices using the DLLME method. Values are reported as a range from three different wines of the same variety at three different spiked concentrations.

		White wines		
**Aroma compound**	**Concentration Level**	**Sauvignon blanc**	**Chardonnay**	**Pinot gris**
Alcohols/Aldehydes				
1-butanol	Low	85.4– 117.4	89.2 - 115.6	81.9 - 111.4
	Medium	97.3 – 108.2	95.5 - 115.9	84.7 - 118.2
	High	86.1 - 115.9	94.9 - 109.7	82.1 - 102.2
benzaldehyde	Low	82.3 - 101.8	88.8 - 118.2	88.9 - 105.4
	Medium	99.5 - 116.2	99.7 - 118.1	83.4 - 106.9
	High	89.9 - 113.4	81.3 - 107.5	86.0 - 119.6
benzyl alcohol	Low	81.2 - 112.6	94.6 - 116.0	89.5 - 101.1
	Medium	86.9 - 118.7	86.5 - 111.0	102.6 - 119.1
	High	106.7 - 109.5	97.0 - 113.5	96.4 - 116.7
isoamyl alcohol	Low	109.7 - 117.8	104.2 - 119.4	85.4 - 113.4
	Medium	92.5 - 108.6	86.2 - 112.0	89.6 - 119.2
	High	82.7 - 111.6	104.5 - 119.4	97.2 - 114.1
isobutanol	Low	82.6 - 119.1	92.7 - 118.9	98.1 - 117.1
	Medium	88.3 - 116.8	100.3 - 116.1	102.1 - 117.6
	High	88.2 - 113.7	89.5 - 118.9	101.5 - 113.6
methionol	Low	94.6 - 115.0	93.4 - 117.8	97.3 - 108.2
	Medium	95.3 - 107.7	89.7 - 113.7	87.7 - 119.9
	High	92.7 - 112.5	99.5 - 112.6	100.6 - 120.0
phenylethyl alcohol	Low	93.2 - 118.7	94.2 - 115.2	99.0 - 117.0
	Medium	107.5 - 120.0	85.8 - 118.7	94.2 - 106.9
	High	91.9 - 116.6	95.3 - 108.6	96.4 - 112.7
C6 compounds				
1-hexanol	Low	105.8 - 118.6	93.5 - 111.9	85.9 - 110.3
	Medium	87.1 - 118.2	104.5 - 116.2	109.0 - 117.6
	High	96.8 - 119.9	90.6 - 95.2	87.2 - 118.0
cis-2-hexen-1-ol	Low	88.3 - 113.5	91.8 - 110.4	113.2 - 116.4
	Medium	91.9 - 104.4	104.6 - 110.3	95.6 - 114.6
	High	85.4 - 113.0	106.4 - 119.8	106.1 - 118.2
cis-3-hexen-1-ol	Low	101.3 - 112.3	94.9 - 109.3	113.1 - 116.8
	Medium	81.3 - 102.1	87.9 - 118.1	102.8 - 117.3
	High	95.3 - 118.4	100.2 - 116.2	110.7 - 120.0
hexanal	Low	90.1 - 110.6	100.8 - 118.1	113.0 - 119.0
	Medium	85.6 - 105.7	95.9 - 113.9	98.9 - 118.7
	High	92.2 - 114.2	98.0 - 118.5	118.9 - 120.0
trans-2-hexen-1-ol	Low	102.7 - 116.7	91.4 - 113.4	102.2 - 111.8

	Medium	88.8 - 112.3	102.5 - 119.7	110.7 - 119.0
	High	85.9 - 110.1	93.1 - 111.0	98.9 - 116.1
trans-2-hexenal	Low	87.5 - 106.7	104.9 - 119.1	98.8 - 109.3
	Medium	88.1 - 113.3	90.6 - 110.2	100.9 - 115.5
	High	95.9 - 113.7	107.5 - 117.1	89.6 - 113.9
trans-3-hexen-1-ol	Low	101.0 - 120.5	93.6 - 116.5	88.3 - 112.7
	Medium	105.5 - 117.9	105.7 - 117.8	104.1 - 115.6
	High	94.1 - 114.9	106.8 - 110.0	87.5 - 118.8
Esters				
ethyl decanoate	Low	92.7 - 113.6	88.1 - 109.9	98.6 - 109.6
	Medium	102.9 - 118.7	88.6 - 113.0	88.5 - 114.6
	High	86.9 - 118.8	100.4 - 119.1	105.8 - 116.0
ethyl hexanoate	Low	111.6 - 118.0	91.5 - 118.2	105.2 - 115.2
	Medium	81.7 - 112.3	96.8 - 119.7	87.6 - 103.6
	High	99.5 - 117.1	96.3 - 104.1	83.4 - 118.5
ethyl octanoate	Low	81.8 - 89.9	84.7 - 106.9	96.2 - 119.0
	Medium	95.6 - 118.0	94.5 - 113.3	89.7 - 107.5
	High	98.2 - 119.7	85.9 - 108.7	95.7 - 118.8
ethyl phenyl acetate	Low	84.5 - 111.4	93.6 - 115.0	97.2 - 119.2
	Medium	100.0 - 109.6	99.9 - 115.2	81.8 - 111.1
	High	104.5 - 119.0	106.2 - 118.5	112.7 - 116.8
hexyl acetate	Low	107.3 - 112.2	96.6 - 119.3	110.8 - 117.9
	Medium	104.5 - 119.9	85.7 - 109.6	95.8 - 106.2
	High	88.1 - 102.6	102.6 - 113.6	105.1 - 118.1
isoamyl acetate	Low	85.5 - 120.0	101.2 - 115.5	97.8 - 111.7
	Medium	105.1 - 116.1	97.5 - 111.5	95.7 - 108.5
	High	85.6 - 116.2	104.5 - 117.1	95.8 - 115.1
β-phenylethyl acetate	Low	95.3 - 114.8	111.4 - 119.3	97.1 - 117.8
	Medium	105.8 - 113.0	90.4 - 116.2	113.0 - 119.3
	High	87.1 - 110.8	107.6 - 118.7	95.4 - 108.7
Fatty acids				
decanoic acid	Low	85.2 - 104.8	100.6 - 116.5	87.1 - 119.6
	Medium	94.8 - 110.4	85.7 - 116.7	94.2 - 117.0
	High	86.5 - 113.1	102.3 - 116.5	103.3 - 117.4
hexanoic acid	Low	102.7 - 119.6	88.2 - 108.5	110.5 - 119.2
	Medium	88.8 - 116.9	104.4 - 117.3	86.1 - 111.0
	High	85.9 - 116.9	98.2 - 114.5	100.7 - 115.7
isobutyric acid	Low	86.5 - 116.1	96.6 - 107.1	86.3 - 106.2
	Medium	97.4 - 116.8	107.2 - 119.0	107.3 - 119.3
	High	89.1 - 108.0	98.4 - 112.4	86.5 - 116.4
isovaleric acid	Low	98.2 - 99.4	86.2 - 119.2	82.6 - 118.8
	Medium	90.8 - 115.3	105.0 - 117.3	90.6 - 112.4

	High	91.4 - 112.9	86.4 - 107.3	102.7 - 119.3
octanoic acid	Low	87.1 - 109.1	104.2 - 117.6	98.6 - 116.4
	Medium	109.9 - 114.2	91.1 - 107.3	89.2 - 117.0
	High	97.7 - 107.9	91.1 - 118.5	87.5 - 119.1
C13-Norisoprenoids/Terpenes				
geraniol	Low	103.8 - 118.6	103.0 - 116.8	107.4 - 116.3
	Medium	97.0 - 106.4	96.5 - 113.2	88.3 - 105.3
	High	106.2 - 111.9	101.1 - 109.2	98.0 - 113.5
linalool	Low	103.5 - 118.5	101.0 - 118.6	89.5 - 100.0
	Medium	106.9 - 116.5	88.2 - 110.4	99.4 - 119.2
	High	115.6 - 118.9	106.3 - 115.6	111.5 - 119.5
nerol	Low	106.4 - 118.1	92.7 - 111.5	114.5 - 119.8
	Medium	89.8 - 111.1	102.9 - 113.8	94.3 - 116.8
	High	92.4 - 111.6	97.5 - 117.0	97.3 - 110.2
α-ionone	Low	95.6 - 119.4	104.6 - 109.8	96.6 - 111.8
	Medium	102.5 - 113.8	97.2 - 120.0	105.7 - 115.4
	High	82.5 - 119.1	86.2 - 113.2	86.3 - 115.2
α-terpineol	Low	95.0 - 108.5	90.0 - 104.8	112.2 - 118.3
	Medium	107.8 - 112.7	101.3 - 117.4	115.2 - 120.0
	High	94.4 - 113.7	96.3 - 116.1	100.8 - 113.6
β-citronellol	Low	103.7 - 117.0	88.2 - 117.5	92.7 - 97.1
	Medium	86.9 - 104.5	104.9 - 110.2	87.9 - 109.7
	High	94.1 - 112.1	87.0 - 108.3	103.1 - 112.3
β-damascenone	Low	92.0 - 112.5	96.0 - 114.2	99.0 - 119.1
	Medium	101.3 - 105.4	101.8 - 115.3	102.1 - 115.1
	High	107.1 - 116.2	110.6 - 119.3	103.4 - 119.0
β-ionone	Low	85.2 - 114.2	98.6 - 111.7	85.4 - 115.1
	Medium	88.5 - 110.2	105.5 - 116.3	97.0 - 108.7
	High	96.6 - 119.2	101.3 - 108.2	86.4 - 119.2
Volatile Phenols				
4-ethyl guaiacol	Low	106.0 - 116.6	110.6 - 117.7	113.9 - 119.4
	Medium	91.3 - 107.3	98.6 - 113.9	101.6 - 115.5
	High	93.8 - 118.2	88.7 - 118.0	108.9 - 118.0
4-ethyl phenol	Low	88.4 - 119.0	85.2 - 119.5	94.8 - 111.1
	Medium	104.7 - 116.1	86.6 - 113.9	85.2 - 117.3
	High	103.4 - 109.9	100.5 - 119.5	98.3 - 116.7

		Red wines		
Aroma compound	Concentration Level	Syrah	Pinot noir	Merlot
Alcohols/Aldehydes				
1-butanol	Low	85.3 - 119.2	89.3 - 112.2	92.9 - 102.8
	Medium	84.6 - 109.4	85.7 - 107.6	91.5 - 118.3
	High	94.2 - 118.5	92.2 - 108.3	91.2 - 108.2
benzaldehyde	Low	87.5 - 103.1	86.3 - 105.0	97.7 - 112.7
	Medium	101.3 - 116.7	90.2 - 117.5	89.6 - 105.5
	High	82.6 - 117.2	86.5 - 118.0	91.9 - 114.9
benzyl alcohol	Low	85.8 - 111.3	86.5 - 109.9	88.7 - 110.4
	Medium	92.4 - 113.6	91.3 - 100.4	95.3 - 117.6
	High	90.6 - 107.2	86.0 - 114.4	96.7 - 119.4
isoamyl alcohol	Low	109.5 - 117.9	94.5 - 118.1	87.7 - 116.2
	Medium	91.8 - 115.9	88.8 - 120.0	96.4 - 115.0
	High	107.1 - 120.2	87.8 - 115.3	95.9 - 108.5
isobutanol	Low	89.5 - 106.1	100.4 - 110.9	84.7 - 112.5
	Medium	98.7 - 118.4	113.3 - 119.0	92.4 - 118.2
	High	96.7 - 108.9	112.8 - 116.6	84.5 - 114.8
methionol	Low	96.8 - 120.0	94.9 - 111.4	81.6 - 113.3
	Medium	85.8 - 115.3	87.1 - 101.7	80.2 - 116.0
	High	89.1 - 112.6	85.9 - 108.3	80.6 - 102.4
phenylethyl alcohol	Low	85.5 - 117.3	99.2 - 117.7	80.7 - 119.5
	Medium	101.9 - 102.6	88.8 - 118.1	82.7 - 115.0
	High	88.8 - 108.1	97.3 - 104.0	84.2 - 106.9
C6 compounds				
1-hexanol	Low	108.8 - 116.2	86.9 - 112.4	84.7 - 110.1
	Medium	95.9 - 115.3	84.6 - 115.5	96.1 - 102.2
	High	89.1 - 111.1	87.5 - 110.5	111.2 - 117.7
cis-2-hexen-1-ol	Low	93.7 - 113.9	88.4 - 112.0	87.1 - 116.8
	Medium	86.3 - 111.9	84.2 - 119.1	88.1 - 119.1

	High	98.3 - 108.2	86.1 - 108.1	88.9 - 111.3
cis-3-hexen-1-ol	Low	103.6 - 118.7	87.8 - 117.6	94.8 - 100.7
	Medium	91.8 - 116.7	80.7 - 118.8	95.6 - 115.0
	High	100.2 - 116.8	86.3 - 118.0	87.8 - 119.5
hexanal	Low	90.7 - 114.1	83.3 - 95.7	94.9 - 102.7
	Medium	84.7 - 116.9	89.6 - 119.0	89.8 - 117.9
	High	103.4 - 119.4	81.1 - 103.2	95.5 - 114.6
trans-2-hexen-1-ol	Low	100.3 - 119.1	87.6 - 107.2	106.8 - 120.0
	Medium	99.3 - 102.4	92.3 - 107.5	87.5 - 108.1
	High	99.3 - 118.6	96.3 - 109.3	89.3 - 110.6
trans-2-hexenal	Low	94.4 - 112.9	89.4 - 112.6	85.4 - 106.4
	Medium	89.0 - 107.1	112.3 - 119.1	97.3 - 110.9
	High	89.4 - 104.7	98.5 - 118.7	86.0 - 112.7
trans-3-hexen-1-ol	Low	90.3 - 116.3	109.9 - 119.1	90.8 - 113.0
	Medium	115.2 - 119.1	106.0 - 115.6	88.2 - 106.7
	High	108.1 - 119.8	104.6 - 117.0	87.4 - 95.6
Esters				
ethyl decanoate	Low	100.4 - 119.0	95.8 - 120.0	94.8 - 101.8
	Medium	111.8 - 118.5	86.5 - 115.1	97.3 - 114.1
	High	89.4 - 119.9	92.9 - 108.8	92.0 - 116.0
ethyl hexanoate	Low	100.1 - 117.4	94.1 - 114.5	85.7 - 106.8
	Medium	114.4 - 118.6	85.6 - 110.8	88.9 - 102.6
	High	104.0 - 119.9	111.5 - 118.3	85.4 - 114.3
ethyl octanoate	Low	111.7 - 116.9	96.0 - 113.8	89.4 - 111.7
	Medium	89.8 - 107.6	85.3 - 119.2	81.4 - 107.2
	High	88.3 - 116.3	88.2 - 116.4	89.2 - 115.9
ethyl phenyl acetate	Low	103.8 - 116.2	118.7 - 119.0	81.1 - 119.7
	Medium	102.8 - 117.9	109.6 - 117.4	97.3 - 117.6
	High	111.0 - 116.6	113.4 - 118.7	82.2 - 118.1
hexyl acetate	Low	98.7 - 117.3	87.3 - 120.0	85.5 - 100.2
	Medium	108.5 - 116.5	100.9 - 116.5	90.0 - 118.4

	High	89.1 - 107.8	88.4 - 119.7	85.7 - 111.9
isoamyl acetate	Low	112.2 - 118.8	114.2 - 118.3	85.6 - 97.5
	Medium	101.5 - 119.3	107.0 - 119.8	92.5 - 103.5
	High	101.7 - 109.2	109.0 - 116.0	85.5 - 97.4
β-phenylethyl acetate	Low	106.3 - 119.3	98.2 - 119.2	98.5 - 119.5
	Medium	111.7 - 116.9	106.5 - 118.1	95.2 - 116.8
	High	85.6 - 109.9	112.8 - 119.5	85.0 - 119.0
Fatty acids				
decanoic acid	Low	112.1 - 120.0	87.5 - 105.3	98.9 - 117.0
	Medium	116.8 - 118.4	89.3 - 106.3	113.2 - 116.1
	High	85.6 - 108.8	87.1 - 109.7	94.4 - 106.7
hexanoic acid	Low	101.0 - 118.1	99.0 - 113.3	117.5 - 120.0
	Medium	87.1 - 112.0	89.3 - 107.3	105.8 - 120.0
	High	98.8 - 119.0	88.0 - 102.9	103.9 - 119.5
isobutyric acid	Low	88.5 - 115.9	89.2 - 120.0	90.8 - 113.2
	Medium	89.0 - 114.4	109.6 - 116.1	85.5 - 118.1
	High	87.6 - 119.4	81.7 - 118.6	98.4 - 109.6
isovaleric acid	Low	86.0 - 108.7	88.1 - 119.6	89.0 - 104.3
	Medium	96.0 - 120.0	88.9 - 102.3	89.4 - 114.7
	High	93.1 - 111.5	88.3 - 117.0	94.4 - 117.2
octanoic acid	Low	94.6 - 116.8	88.2 - 101.9	102.0 - 116.5
	Medium	81.9 - 117.6	95.2 - 113.5	102.4 - 116.2
	High	87.8 - 111.4	97.0 - 110.7	115.9 - 120.0
C13-Norisoprenoids/Terpenes				
geraniol	Low	91.0 - 103.9	88.2 - 116.2	111.9 - 119.3
	Medium	85.4 - 117.2	89.5 - 116.3	103.0 - 118.9
	High	91.4 - 118.6	107.2 - 119.3	94.1 - 119.5
linalool	Low	84.1 - 113.9	96.1 - 119.5	87.5 - 119.7
	Medium	101.2 - 119.7	89.8 - 113.8	88.6 - 107.8
	High	89.1 - 118.5	84.7 - 115.4	89.1 - 102.5
nerol	Low	93.0 - 115.0	85.1 - 109.8	87.5 - 106.7

	Medium	107.3 - 118.1	90.8 - 106.3	92.5 - 116.9
	High	93.0 - 107.4	99.7 - 120.0	111.0 - 118.2
α-ionone	Low	85.7 - 103.2	105.0 - 109.9	88.5 - 100.9
	Medium	102.0 - 118.2	86.8 - 113.5	96.1 - 116.6
	High	87.5 - 115.6	93.2 - 109.8	99.5 - 104.1
α-terpineol	Low	94.6 - 119.2	89.3 - 118.2	85.3 - 120.0
	Medium	86.0 - 119.1	96.6 - 109.9	89.6 - 115.8
	High	93.4 - 119.8	94.0 - 117.7	85.6 - 114.3
β-citronellol	Low	105.2 - 114.9	105.3 - 112.4	101.7 - 117.6
	Medium	12.9 - 116.9	91.5 - 108.0	90.2 - 109.1
	High	108.5 - 110.4	106.9 - 112.1	97.6 - 106.1
β-damascenone	Low	100.8 - 111.6	89.4 - 116.5	89.4 - 116.0
	Medium	108.6 - 118.4	87.3 - 107.1	87.1 - 120.0
	High	105.4 - 118.5	88.8 - 111.6	88.5 - 111.5
β-ionone	Low	86.8 - 111.8	81.8 - 112.9	89.2 - 112.7
	Medium	84.4 - 106.5	85.7 - 114.8	89.3 - 112.0
	High	87.6 - 116.4	87.1 - 112.5	88.4 - 117.1
Volatile Phenols				
4-ethyl guaiacol	Low	96.7 - 118.0	85.7 - 113.3	87.8 - 114.6
	Medium	91.5 - 117.4	94.7 - 119.3	95.3 - 111.5
	High	90.2 - 111.7	94.5 - 101.0	89.3 - 105.8
4-ethyl phenol	Low	85.8 - 102.2	95.4 - 117.6	87.0 - 116.0
	Medium	87.6 - 116.3	97.7 - 117.9	108.2 - 119.1
	High	84.9 - 106.7	102.4 - 119.2	88.5 - 113.2

Table S8: Details of the 30 NZ PN wines (2020 vintage) included in the method application study.

Region	Wine	Purchase price (NZD) in 2022
Marlborough	Ara PN	$10 to $30
	Allan Scott Estate Black	
	Lake Chalice The Nest	
	Pask Instinct	
	Duck Hunter	
	Saint Clare	Greater than $40
	Villa Maria Reserve	
	Wairau River Reserve	
	Auntsfield Single Vineyard	
	Astrolabe Comelybank Waihopai Valley	
Central Otago	Madam Sass	$10 to $30
	Terra Sancta	
	Rockey Point	
	Mud House	
	Akarua Rua	
	Felton Road Calvert	Greater than $40
	Rockburn Wines	
	Felton Road, Bannockburn	
	Two Paddocks	
	Hawkshead	
Martinborough	Luna Estate	$10 to $30
	Nga Waka Three Paddles	
	Alexander Dusty Road	
	Clearview Estate	
	The Edge by Escarpment	
	Te Kairanga John Martin	Greater than $40
	Nga Waka Lease Block	
	Palliser Estate	
	Caro’s Big Sky	
	Cambridge Road	
